# Supplementary material for: Diversity of tooth mineralisation patterns at the base of crown chondrichthyans
Source: Commun Biol. 2025 Dec 3;9:56. doi: 10.1038/s42003-025-09320-0 (PMC12796304; doi:10.1038/s42003-025-09320-0)
Supplement: Supplementary file 1 — Supplementary Information [file 42003_2025_9320_MOESM1_ESM.pdf]

Supplementary Information for

Diversity of tooth mineralization patterns at the base of crown chondrichthyans

Greif, M., Botella, H., Scheyer, T. M., Klug, C.

Supplementary Note 1. Oral denticles

The oral denticles of *Ct. concinnus* are between 1 and 3 mm wide and are mainly preserved between the tooth bases and the jaw cartilage. Some are also present around the tooth cusps (main text fig. 2 A). The histology consists of hypermineralized enameloid, pallial orthodentine and porous osteodentine (Fig. 1A, B). Generally, there are several main cusps discernible as typical for polyodontode denticles (about 4 -5, Fig. 1A, B; see also supplementary online material at Zenodo: 10.5281/zenodo.15387379).

The polyodontode oral denticles of *P. saidselachus* are up to 1 mm wide. They are scattered in the matrix around the teeth (main text fig. 2B). Highly mineralized enameloid caps the cusps of the denticles (Fig. 1C, D, see also supplementary online material at Zenodo: 10.5281/zenodo.15387379).

The oral denticles of *M. mohamezanei* are between 1 and 2.5 mm wide. They are aligned along the upper and lower jaw cartilage and occasionally scattered between the teeth (main text fig. 2G, H). The denticles are made of orthodentine. More histological details are not discernible. They are polyodontode and up to five cusps can be seen (Fig. 7E, F, see also supplementary online material at Zenodo: 10.5281/zenodo.15387379).

Supplementary Figure 1

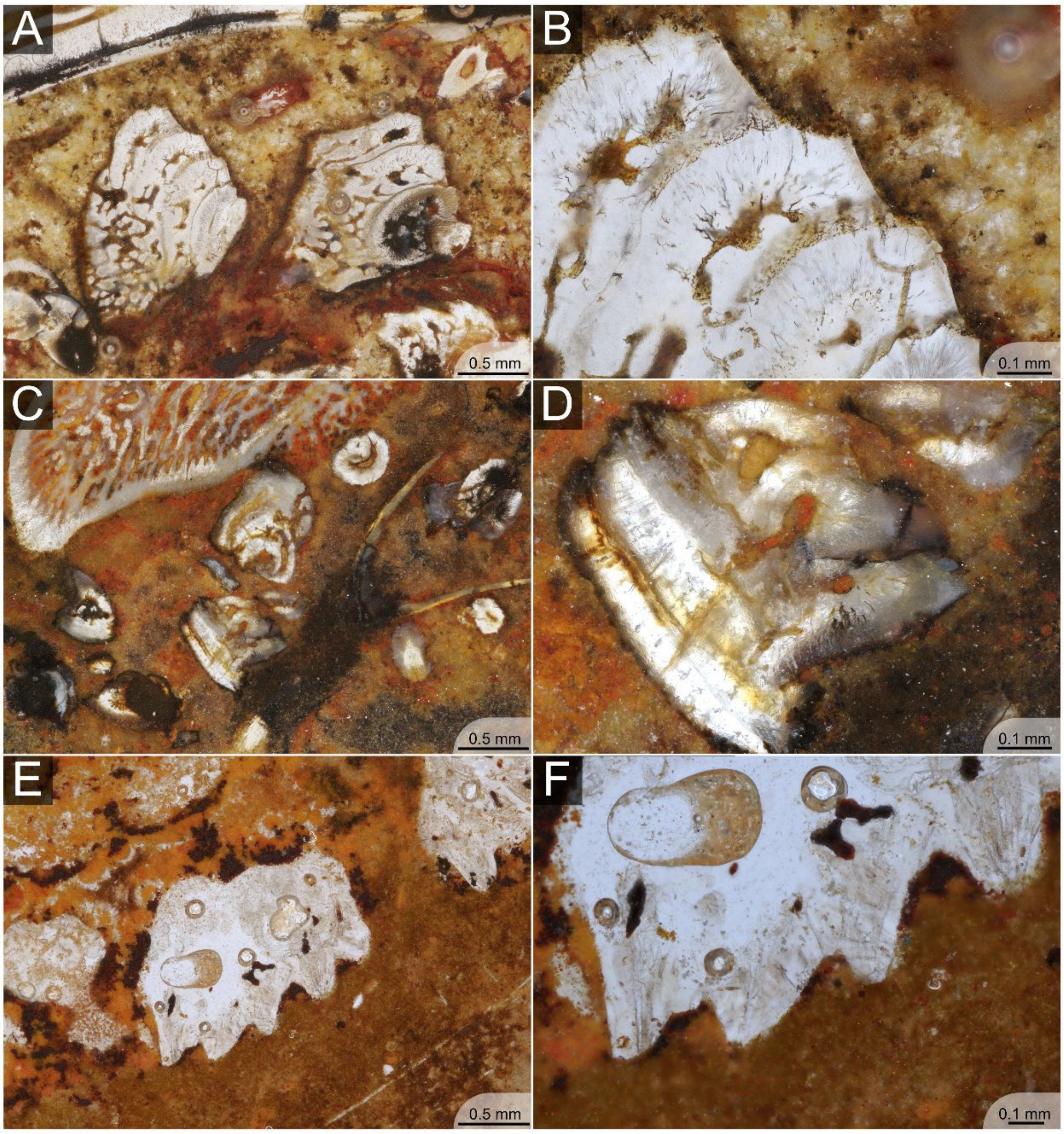

Figure 1: Oral denticles. A, B: PIMUZ A/I 5304, *Ctenacanthus concinnus*; C, D: AA.MEM.DS.2, *Phoebodus saidselachus*. E, F: PIMUZ A/I 5306, *Maghriboselache mohamezanei. For the original* high-quality photos and further images, see also supplementary *online* material at Zenodo: 10.5281/zenodo.15387379).

Supplementary Note 2. Mineralization sequence of the teeth of Squatina

The mineralization sequence of the teeth of *Squatina squatina* shows striking similarities with the patterns in *Phoebodus saidselachus* (main text). In both, orthodentine is not restricted to the crown but migrates in early development down to the base. However, *Squatina* does not preserve a hollow pulp cavity like *Phoebodus*. This pattern shows that orthodentine and osteodentine develops from different odontoblast cell populations that follows different

histogenetic trajectories, as firstly suggested by Peyer[2].Thus, orthodentine deposited from mesenchymal cells differentiates into odontoblast in the periphery of the tooth ("peripheral initial zone" *sensu* Peyer[2]). As the odontoblasts retreat centripetally toward the centre of the pulp cavity, they leave behind an increasingly thick layer of orthodentine decreasing the surface of the pulp cavity. Conversely, osteodentine has an intrapulpar origin [2,3]. Thus, "scleroblasts" that produce osteodentine differentiate inside the dental papilla (the *"sudden appearance"* of osteodentine *sensu* Peyer[2]; see Moyer et al.[3]). They appear in the developing tooth between adjacent vessels depositing dentine while the cells retreat towards their respective vessels forming concentric cylinders of osteodentine[2]. Usually, separate, not synchronized, populations of osteodentine-producing odontoblasts can appear within the pulp cavity such as in *Carcharodon carcharias*[3] or in *Squatina* (Fig. 3).

Supplementary Figure 2

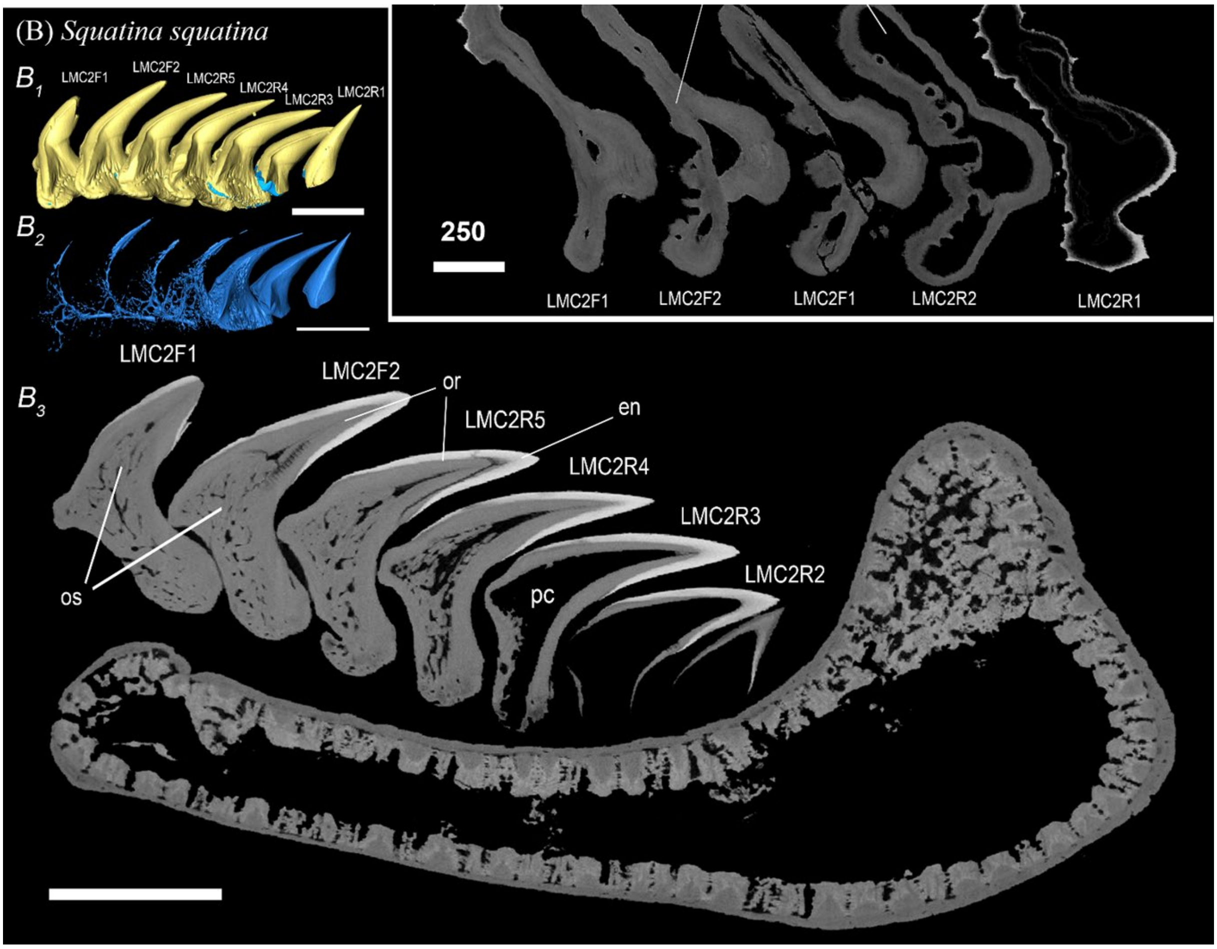

Figure 2: Tooth file of *Squatina squatina* showing a similar mineralization pattern as Phoebodus. Naming of the teeth follows Moyer et al. [3]: "Left, Meckel's cartilage [MC], file number 2. The letter F indicates a functional tooth; the letter R indicates a replacement tooth."

Supplementary Note 3. Size measurements and tooth replacement rates

We estimated the rate of tooth replacement for *Maghriboselache mohamezanei* based on an approach proposed by Botella et al.[1]. They provide a numerical framework in which the tooth size increment (Δs) within consecutive teeth of the same dental file is used as an approximation for the speed of tooth replacement in fossil taxa (in days/row). Replacement rates were estimated using the following equation:

$$\Delta s = \frac{\left[\sum \frac{(width\ tooth\ (T) - width\ tooth\ (T-1))}{width\ tooth(T-1)}\right]}{N\ obs.} \quad \text{(Botella et al.}^{1}\text{)}$$

Δs = (in %) Average increment in the maximum widths between consecutive teeth of the same tooth family.

Nobs = number of observations (differences made)

Measurements of tooth width of individual teeth of a single tooth file (Fig. 2) as given below (in mm):

File 1 : T1 = 5.04; T2 = 5.95; T3 = 7.41; T4 = 7.80; T5 = 8.21; T6 = 9.62; T7 = 9.89; T8 = 11.0;

Δs = 12.04

Note. - The bases of teeth T9 to T 11 (the youngest) are not fully developed, and therefore not considered in the measurements.

Supplementary Figure 3

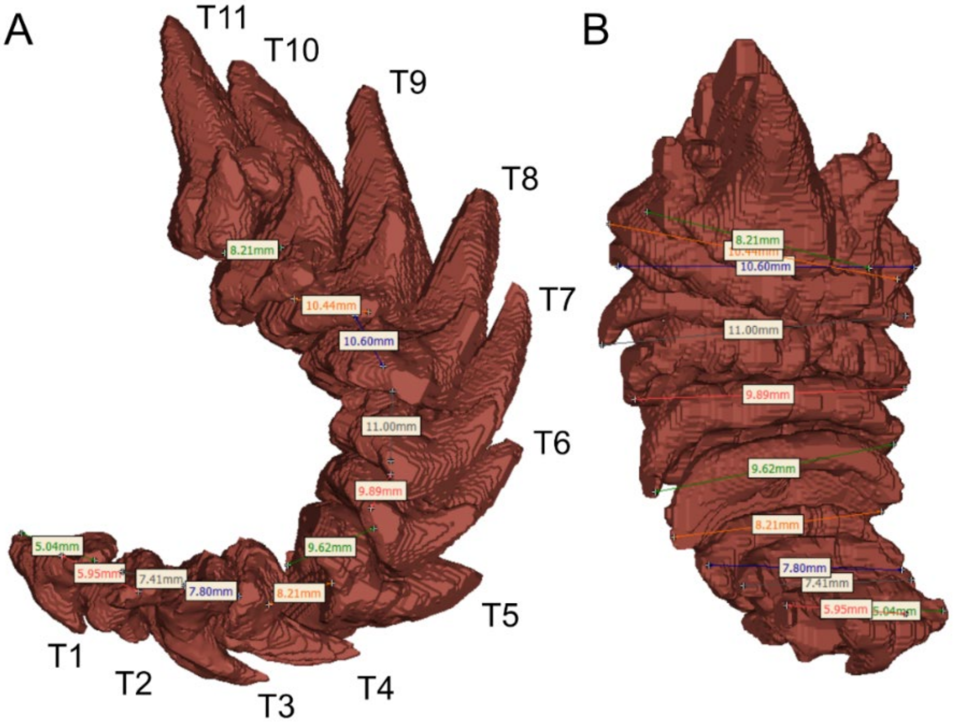

Figure 3: 3D reconstruction of a single tooth file of Maghriboselache, from CT images (File 9-10, *10.5281/zenodo.15387379)* (named as File1 above) showing base width measurements for all teeth of a proximate tooth file of the Meckel's cartilage of *M. mohamezanei*

Supplementary Table 1:

Presence and absence of dentine types in Palaeozoic chondrichthyans: List of all taxa that we have been able to find information regarding histology within the entire tooth. For each the presence of the different types of dentine/ enameloid are shown for crown and base as well as the presence or absence of a pulp cavity. ena = enameloid; orth = orthodentine; trd = trabecular dentine.

Taxa	Crown	Base	Pulp cavity
**Phoebodontidae**			
Phoebodus (saidselachus), (this study)	ena + orth + trd	orth + trd	yes, small
**Ctenacanthidae**			
Ctenacanthus (concinnus), (this study)	ena + orth + trd	trd	no
Ctenacanthus (costellatus), [4]	ena + orth + trd	trd	no
**Cladoselachidae**			
Maghriboselache (mohamezanei) (This study)	ena + orth + trd	trd	no
Cladoselache [5]	ena + orth (thick) + trd	trd (presumed)	no
**Symmoriidae**			
Symmorium [4]	ena + orth + trd	trd	no
Stethacanthus [4]	ena+ orth + trd	trd	no
**Sister group all remaining chondrichthyans**			
Leonodus (carlsi)[6]	ena + orth + trd	trd	no
**Xenacanthidae**			
Antarctilamma (prisca) [4]	orth (thick) + osdt	trd	no
Reginaselache (morrisi) [7]	orth + trd	orth + trd	no
Mooreodontus indicus[8]	orth	trd	yes, small
Omalodontiformes			
Omalodontidae			
Portalodus (bradshawae) [4]	ena (very thin) + orth (thin) + trd	trd	no
Aztecodontidae			
Aztecodus (harmsenae) [4]	pleromin	osdt	no

Supplementary References

1. Botella, H., Valenzuela-Ríos, J. I. & Martínez-Pérez, C. Tooth replacement rates in early chondrichthyans: a qualitative approach. *Lethaia* **42**, 365–376 (2009).
2. Peyer, B. Comparative Odontology. *The University of Chicago* (1968).
3. Moyer, J., Riccio, M. & Bemis, W. Development and microstructure of tooth histotypes in the blue shark, *Prionace glauca* (Carcharhiniformes: Carcharhinidae) and the great white shark, *Carcharodon carcharias* (Lamniformes: Lamnidae): *J. Morphol.* **276**, 797-817 (2015).
4. Hampe, O. & Long, J. The histology of Middle Devonian chondrichthyan teeth from southern Victoria Land, Antarctica. *Records of the Western Australian Museum* **57**, 23–36 (1999).
5. Gillis, J. A. & Donoghue, P. C. J. The homology and phylogeny of chondrichthyan tooth enameloid. *J. Morphol.* **268**, 33–49 (2007).
6. Botella, H., Donoghue, P. C. J. & Martínez-Pérez, C. Enameloid microstructure in the oldest known chondrichthyan teeth. *Acta Zool.* **90**, 103–108 (2009).
7. Turner, S. & Burrow, C. J. A Lower Carboniferous xenacanthiform shark from Australia. *J. Vertebr. Paleontol.* **31**, 241–257 (2011).
8. Bhat, M. S., Ray, S. & Datta, P. M. A new assemblage of freshwater sharks (Chondrichthyes: Elasmobranchii) from the Upper Triassic of India. *Geobios* **51**, 269–283 (2018).
