# Supplementary material for: Diversity of tooth mineralisation patterns at the base of crown chondrichthyans
Source: Commun Biol. 2025 Dec 3;9:56. doi: 10.1038/s42003-025-09320-0 (PMC12796304; doi:10.1038/s42003-025-09320-0)
Supplement: Supplementary file 2 — Reporting Summary [file 42003_2025_9320_MOESM2_ESM.pdf]

nature portfolio

Corresponding author(s): Merle Greif
Department of Palaeontology, University of Zurich
merle.greif@pim.uzh.ch

Last updated by author(s): Nov 20, 2025

# Reporting Summary

Nature Portfolio wishes to improve the reproducibility of the work that we publish. This form provides structure for consistency and transparency in reporting. For further information on Nature Portfolio policies, see our Editorial Policies and the Editorial Policy Checklist.

## Statistics

For all statistical analyses, confirm that the following items are present in the figure legend, table legend, main text, or Methods section.

| n/a | Confirmed | |
|---|---|---|
| ☐ | ☒ | The exact sample size (*n*) for each experimental group/condition, given as a discrete number and unit of measurement |
| ☐ | ☒ | A statement on whether measurements were taken from distinct samples or whether the same sample was measured repeatedly |
| ☒ | ☐ | The statistical test(s) used AND whether they are one- or two-sided<br>*Only common tests should be described solely by name; describe more complex techniques in the Methods section.* |
| ☒ | ☐ | A description of all covariates tested |
| ☒ | ☐ | A description of any assumptions or corrections, such as tests of normality and adjustment for multiple comparisons |
| ☒ | ☐ | A full description of the statistical parameters including central tendency (e.g. means) or other basic estimates (e.g. regression coefficient) AND variation (e.g. standard deviation) or associated estimates of uncertainty (e.g. confidence intervals) |
| ☒ | ☐ | For null hypothesis testing, the test statistic (e.g. *F*, *t*, *r*) with confidence intervals, effect sizes, degrees of freedom and *P* value noted<br>*Give P values as exact values whenever suitable.* |
| ☒ | ☐ | For Bayesian analysis, information on the choice of priors and Markov chain Monte Carlo settings |
| ☒ | ☐ | For hierarchical and complex designs, identification of the appropriate level for tests and full reporting of outcomes |
| ☒ | ☐ | Estimates of effect sizes (e.g. Cohen's *d*, Pearson's *r*), indicating how they were calculated |

*Our web collection on statistics for biologists contains articles on many of the points above.*

## Software and code

Policy information about availability of computer code

| | |
|---|---|
| Data collection | no software was used |
| Data analysis | no software was used |

For manuscripts utilizing custom algorithms or software that are central to the research but not yet described in published literature, software must be made available to editors and reviewers. We strongly encourage code deposition in a community repository (e.g. GitHub). See the Nature Portfolio guidelines for submitting code & software for further information.

## Data

Policy information about availability of data

All manuscripts must include a data availability statement. This statement should provide the following information, where applicable:

- Accession codes, unique identifiers, or web links for publicly available datasets
- A description of any restrictions on data availability
- For clinical datasets or third party data, please ensure that the statement adheres to our policy

Thin sections of the specimens ESEFB-LTM-201, Ctenacanthus concinnus, ESEFB-LTM-202, Maghriboselache mohamezanei and ESEFB-LTM-203, Phoebodus saidselachus, are stored in the collections of the Higher School of Education and Training Berrechid, Hassan First University, Berrechid, Morocco.
Supplementary Information includes Supplementary Notes 1-3 and accompanying figures. Supplementary Information 1: Note on oral denticles and Figure depicting these in the thin sections of all three specimens. Supplementary Figure 1: Histology of the oral denticles of all three specimens Supplementary Information 2: Note

on the mineralization sequence of the teeth of Squatina. Supplementary Figure2: Tooth file of Squatina. Supplementary Information 3: Note on size measurements and tooth replacement rates including the equation used to estimate replacement for Maghriboselache. Supplementary Figure 3: 3D reconstruction of a tooth file of Maghriboselache showing base width measurements. Supplementary Data (File 1-10) includes unprocessed images and the CT-data as well as 3D reconstructions of the specimens, stored on Zenodo: 10.5281/zenodo.15387379.

## Research involving human participants, their data, or biological material

Policy information about studies with human participants or human data. See also policy information about sex, gender (identity/presentation), and sexual orientation and race, ethnicity and racism.

| | |
|---|---|
| Reporting on sex and gender | This information has not been collected |
| Reporting on race, ethnicity, or other socially relevant groupings | This information has not been collected |
| Population characteristics | see above |
| Recruitment | see above |
| Ethics oversight | see above |

Note that full information on the approval of the study protocol must also be provided in the manuscript.

# Field-specific reporting

Please select the one below that is the best fit for your research. If you are not sure, read the appropriate sections before making your selection.

☐ Life sciences ☐ Behavioural & social sciences ☒ Ecological, evolutionary & environmental sciences

For a reference copy of the document with all sections, see nature.com/documents/nr-reporting-summary-flat.pdf

# Ecological, evolutionary & environmental sciences study design

All studies must disclose on these points even when the disclosure is negative.

| | |
|---|---|
| Study description | The study is based on histological data of thin sections of Devonian chondrichthyan teeth from Morocco preserved in files. Due to the in situ preservation, it is possible to focus on tooth mineralization patterns of Paleozoic chondrichthyans for the first time. Additionally, CT-data is used to reconstruct not only the three-dimensional properties of the tooth files, but also to draw inferences about tooth replacement rates. The gathered information is discussed in a phylogenetic context. |
| Research sample | The study includes samples of three different taxa of Devonian chondrichthyans: Ctenacanthus concinnus, Maghriboselache mohamezanei and Phoebodus saidselachus. Samples were taken of one specimen of each taxon. Each specimen was CT-scanned and the data included. Between 1-3 histological sections were taken for each specimen. |
| Sampling strategy | Samples were taken based on the outstanding quality of fossilization. Samples were taken along the tooth files including all preserved members of one file. Since the preparation of histological sections is destructive, CT-scans have been performed beforehand to preserve 3D information. |
| Data collection | Histological sections have been prepared by Merle Greif, Torsten Scheyer and partly by Stefanie Herter. Pictures have been taken by Merle Greif. CT-data was obtained using the Industrial CT-Scanner at Qualitech in Mägenwil, Switzerland operated by Pedro Aquino. Segmentation has been performed in Mimics 26.0 by Merle Greif, Irina Valeria Etter and Lena Sophie Gersbach (part of their student internships at the University of Zurich). |
| Timing and spatial scale | Data collection has started in spring 2024. Thin sections were prepared subsequently and pictures taken. Interpretation of the prepared samples started in Summer/autumn 2024. |
| Data exclusions | no data was excluded. |
| Reproducibility | The study is based on three specimens and 1 – 3 thin sections of each, which are stored in the collections of the Higher School of Education and Training Berrechid, Hassan First University, Berrechid, Morocco. Detailed photographs that are not figured in the manuscript are provided online: 10.5281/zenodo.15387379 and complement the figures and histological interpretations ensuring the reproducability of the study. |
| Randomization | This is not relevant to our study. |
| Blinding | This is not relevant to our study. |

Did the study involve field work? ☒ Yes ☐ No

## Field work, collection and transport

| | |
|---|---|
| Field conditions | Field work was carried out in the Anti-Atlas of Morocco in spring time at temperatures about 25-27°C, arid climate. |
| Location | Samples are collected in different localities of the Anti-Atlas. ESEFB-LTM-201, Ctenacanthus concinnus, was found in the Tafilalt at 31°00'57.4"N 4°03'58.2"W; ESEFB-LTM-203 Phoebodus saidselachus was found at Tizi Mouzgar at 30.76937°N, 4.70647°W; ESEFB-LTM-202, Maghriboselache mohamezanei was found in the Tafilalt at 30°57'40.2" 4°02'36.1"W. All fossils are found in iron rich carbonates within the desert. |
| Access & import/export | Sites are accessed by car. The exact sites can only be entered by foot.<br>Work is permitted by the Ministère de l'Energie, des Mines, de l'Eau et de l'Environnement (Direction du Développement Minier, Division du Patrimoine, Rabat, Morocco).<br>All samples are returned to Morocco and housed at Higher School of Education and Training Berrechid (ESEFB), Hassan First University , Berrechid, Morocco. |
| Disturbance | To minimize disturbance, students are adviced to use tools carefully. We furthermore work together with local collaborators (amateur and professional paleontologists). Campsites are carefully cleaned when leaving to avoid littering. |

# Reporting for specific materials, systems and methods

We require information from authors about some types of materials, experimental systems and methods used in many studies. Here, indicate whether each material, system or method listed is relevant to your study. If you are not sure if a list item applies to your research, read the appropriate section before selecting a response.

### Materials & experimental systems

| n/a | Involved in the study | |
|---|---|---|
| ☒ | ☐ | Antibodies |
| ☒ | ☐ | Eukaryotic cell lines |
| ☐ | ☒ | Palaeontology and archaeology |
| ☒ | ☐ | Animals and other organisms |
| ☒ | ☐ | Clinical data |
| ☒ | ☐ | Dual use research of concern |
| ☒ | ☐ | Plants |

### Methods

| n/a | Involved in the study | |
|---|---|---|
| ☒ | ☐ | ChIP-seq |
| ☒ | ☐ | Flow cytometry |
| ☒ | ☐ | MRI-based neuroimaging |

## Palaeontology and Archaeology

| | |
|---|---|
| Specimen provenance | Specimens were collected in the Devonian layers of the Anti-Atlas, Morocco (Tafilalt). Work is permitted by the Ministère de l'Energie, des Mines, de l'Eau et de l'Environnement (Direction du Développement Minier, Division du Patrimoine, Rabat, Morocco); permitted in Rabat, 10. March 2020. Permit N°.1571/DE/DG. |
| Specimen deposition | Thin sections of the specimens ESEFB-LTM-201, Ctenacanthus concinnus, ESEFB-LTM-202, Maghriboselache mohamezanei and ESEFB-LTM-203, Phoebodus saidselachus, are stored in the collections of the Higher School of Education and Training Berrechid, Hassan First University, Berrechid, Morocco. |
| Dating methods | No new dates are provided |

☐ Tick this box to confirm that the raw and calibrated dates are available in the paper or in Supplementary Information.

| | |
|---|---|
| Ethics oversight | not applicable |

Note that full information on the approval of the study protocol must also be provided in the manuscript.

## Plants

| | |
|---|---|
| Seed stocks | not applicable |
| Novel plant genotypes | not applicable |
| Authentication | not applicable |
